# AQP1-Containing Exosomes in Peritoneal Dialysis Effluent As Biomarker of Dialysis Efficiency

**DOI:** 10.3390/cells8040330

**Published:** 2019-04-09

**Authors:** Simone Corciulo, Maria Celeste Nicoletti, Lisa Mastrofrancesco, Serena Milano, Maria Mastrodonato, Monica Carmosino, Andrea Gerbino, Roberto Corciulo, Roberto Russo, Maria Svelto, Loreto Gesualdo, Giuseppe Procino

**Affiliations:** 1 Department of Emergency and Organ Transplantation (DETO), University of Bari, 70124 Bari, Italy; simo.corciulo@gmail.com (S.C.); r.corciulo51@gmail.com (R.C.); roberto.russo@policlinico.ba.it (R.R.); loreto.gesualdo@uniba.it (L.G.); 2 Department of Biosciences, Biotechnologies and Biopharmaceutics, University of Bari, 70125 Bari, Italy; celeste_nicoletti@libero.it (M.C.N.); mastrofrancescolisa@libero.it (L.M.); serena.milano@uniba.it (S.M.); monica.carmosino@unibas.it (M.C.); andrea.gerbino@uniba.it (A.G.); maria.svelto@uniba.it (M.S.); 3 Department of Biology, University of Bari, 70125 Bari, Italy; maria.mastrodonato@uniba.it

**Keywords:** AQP1, peritoneal dialysis, peritoneal equilibrium test, mesothelium

## Abstract

The water channel Aquaporin 1 (AQP1) plays a fundamental role in water ultrafiltration during peritoneal dialysis (PD) and its reduced expression or function may be responsible for ultrafiltration failure (UFF). In humans, AQP1 is expressed in the endothelium of the peritoneal capillaries but its expression in mesothelial cells (MC) and its functional role in PD is still being debated. Here, we studied a cohort of 30 patients using PD in order to determine the presence of AQP1 in peritoneal biopsies, AQP1 release in the PD effluent through exosomes and the correlation of AQP1 abundance with the efficiency of peritoneal ultrafiltration. The experiments using immunofluorescence showed a strong expression of AQP1 in MCs. Immunoblotting analysis on vesicles isolated from PD effluents showed a consistent presence of AQP1, mesothelin and Alix and the absence of the CD31. Thus, this suggests that they have an exclusive mesothelial origin. The immunoTEM analysis showed a homogeneous population of nanovesicles and confirmed the immunoblotting results. Interestingly, the quantitative analysis by ELISA showed a positive correlation between AQP1 in the PD effluent and ultrafiltration (UF), free water transport (FWT) and Na-sieving. This evidence opens the discussion on the functional role of mesothelial AQP1 during PD and suggests that it may represent a potential non-invasive biomarker of peritoneal barrier integrity, with predictive potential of UFF in PD patients.

## 1. Introduction

Peritoneal dialysis (PD) was introduced more than three decades ago as a renal replacement therapy to restore fluid homeostasis in dialysis patients with end-stage renal disease (ESRD).

PD is a simple therapy in which a hypertonic solution containing glucose as its osmotic agent is instilled in the patient’s peritoneal cavity and is exchanged four times a day. The osmotic gradient created between the peritoneal cavity and the plasma allows the removal of excess water. Although PD is cost effective compared to hospital-based hemodialysis [1], only one out ten patients is treated with PD as it has major limitations of peritoneal membrane damage in the long term and increases the risk of infection [2,3]. 

The peritoneum is a continuous membrane that lines the peritoneal cavity. It is composed of a monolayer of mesothelial cells sitting on a basement membrane and a sub-mesothelium characterized by several bundles of collagen fibers, scattered fibroblasts and blood vessels. The continuous exposure of the peritoneum to non-physiological hypertonic PD solutions and occasional peritonitis causes inflammation of the peritoneal membrane, which progressively undergoes fibrosis, angiogenesis and ultimately ultrafiltration failure (UFF). 

The assessment of the PD membrane functionality is usually conducted by investigating the transport of small solutes and fluid using a peritoneal equilibrium test (PET), which was developed and described by Twardowski [4]. PET is based on a dialysis dwell of 4 h using a 2.27–2.5% glucose-based dialysis solution and it measures peritoneal solute transport and water ultrafiltration (UF). The Mini PET test, which was developed by La Milia et al. [5], is a promising tool for assessing free water transport (FWT) and small solute transport across the peritoneal membrane. FWT is calculated after a dwell of 1 h with 3.86% glucose using the kinetics of sodium transport. However, this requires the measurement of intraperitoneal volume after 1 h. Recently, Cnossen et al. [6] proposed a 4-h 3.86% glucose PET, including a temporary drainage after 1 h for the assessment of FWT. This test does not influence the results of small solute transport and provides essential additional information about water UF. Subsequently, this test (two-in-one protocol) was extended to a larger population by simultaneously evaluating both small solute transport and more accurately UF through ultra-small and small pores present on the peritoneal membrane. 

The three-pore model of Rippe and Stelin [7,8] proposed the existence of “ultra-small, water-only pores” (3–5 Å) that are responsible for the phenomenon of sodium sieving during PD. During the early dwell time of the hypertonic PD fluid, the sodium concentration in the dialysate falls as a consequence of the water entering the dialysate from the blood at a faster rate than sodium. Thus, the sodium is apparently “sieved” out of the PD fluid. The ultra-small pores, which were later identified as the water channels Aquaporin 1 (AQP1) [9], mediate about 50% of the water movement during PD with hypertonic crystalloid solutions and could account for the observed dilution of sodium [10]. 

In the peritoneum, AQP1 is expressed in the endothelial cells of peritoneal capillaries and venules but not in arterioles [11,12]. AQP1 expression in the mesothelial epithelium is still debated. It was only found in the endothelial cells of the peritoneum but not in the mesothelial cells [12,13]. On the other hand, a constitutive expression of AQP1 in human peritoneal mesothelial cells was demonstrated by other authors [14,15].

The evaluation of the morphologic and/or metabolic alterations of the peritoneal membrane would require repetitive peritoneal biopsies that usually are not feasible. However, it should be emphasized that peritoneal tissues, bathed in dialysis solutions, may secrete or shed substances, which can be recovered in the peritoneal effluent. These molecular effluents are potential biomarkers that may provide insight into the integrity of the peritoneal membrane and predict the outcome in the ESRD patients. 

At present, there is only modest integration of effluent biomarkers into the routine clinical practice of PD. The only two established biomarkers are cancer antigen 125 (CA125) and interleukin-6 (IL-6), both of which can be measured easily in unconcentrated effluents. Other cytokines and growth factors, along with candidate biomarkers of peritoneum remodeling, coagulation and fibrosis, have been proposed over time [16,17]. Most of them are soluble factors, which does not exclude the possibility that they can be produced elsewhere and are not necessarily always derived from intraperitoneal production. 

In this present work, we confirm that the water channel AQP1 [9] is expressed in the plasma membrane of human mesothelial cells and we demonstrate that the mesothelial AQP1 is released in the PD effluent through exosomes. Moreover, by studying a cohort of 30 patients undergoing a “two-in-one protocol” to quantify small-solute transport, FWT and small-pore ultrafiltration (SPUF) during a single Peritoneal Equilibration Test (PET) procedure, we found a strong positive correlation between the abundance of AQP1 in the PD effluent and the solute and fluid transport parameters that are used to describe the efficiency of PD.

## 2. Materials and Methods 

### 2.1. Patients and Samples Collection

All samples used in this study were collected from thirty patients (19 men and 11 women; mean age: 57 ± 15 years) attending PD Units at Nephrology, Dialysis and Transplant Department of the University Hospital “Policlinico di Bari”, Bari (Italy) between January 2015 and March 2017. The mean time on PD was 24 months (range: 4–79 months). All patients used continuous ambulatory PD (CAPD). None of the patients had peritonitis during the study or the preceding four weeks. The test was performed as a routine annual evaluation of peritoneal membrane status without elective selection. 

Nocturnal peritoneal dialysate samples were collected after an overnight exchange of a PD solution (volume 2 L of glucose 2.27% and lactate/bicarbonate buffer Physioneal Baxter Healthcare Corporation, Deerfield, IL, USA) for 10 h. All the dialysate was drained and the remaining amount was measured. Nocturnal peritoneal dialysate samples were supplemented with Protease Inhibitor Cocktail Tablets (Roche Diagnostics GmbH) to reduce protein degradation, which were subsequently centrifuged at 2000× *g* for 30 min at 4 °C to remove cellular debris before being subjected to the exosome isolation protocol. These samples were used for Western blotting experiments. 

After draining the nocturnal dialysate, the patients underwent a Peritoneal Equilibration Test (PET) modified with a 3.86% glucose hypertonic solution for 4 h, with a temporary drain added at 1 h (two-in-one protocol). This protocol was previously described by Cnossen et al. [6]. Physioneal solutions from Baxter (Baxter Healthcare Corporation, Deerfield, IL, USA) were used during the procedure. A total of 2 L of solution was infused into the peritoneal cavity and after 1 h, the UF volume was measured through the total drainage of the peritoneal cavity. The UF volume was calculated by subtracting the dialysis fluid (fill volume) from the effluent volume and the effluent was immediately reinfused. Blood samples were taken after the complete infusion of the dialysis solution at time 0 and after 60 and 240 min (P0, P60 and P240, respectively). During the first minute after the complete infusion of 2 L dialysis solution and after 60 and 240 min, 10 mL of peritoneal dialysate (D0, D60 and D240, respectively) was sampled. After 240 min, the peritoneal cavity was completely drained and the drained volume was weighed and stored as reported above. These samples were used for AQP1 quantification by ELISA. 

Specimens of human omentum were obtained from three patients undergoing elective abdominal surgery while parietal peritoneal biopsies were collected from three PD patients. 

The study was authorized by the ethics committee at the Department of Emergency and Organ Transplantation (DETO), University of Bari, Italy

All patients provided their signed consent for the use of their tissues for research purposes.

### 2.2. Measurements

Glucose, creatinine and sodium were measured both in blood samples and in dialysate. The Jaffé method was used for creatinine. The creatinine in the dialysate was corrected for glucose interference according to laboratory standards. Sodium concentration both in the dialysate and the plasma was measured with selective ion electrode instruments using an indirect method. Ca125 was also assayed in the dialysate after 4 h using an immunoenzymatic method (Inmulite OM-MA; EURO/DPC Ltd, Lanberis, UK). 

### 2.3. Calculations

The ratios of dialysate glucose concentrations were calculated by dividing the dialysate glucose concentration at the start and after 60 and 240 min (Dt) by the dialysate glucose concentration at the start (D0). The D/P ratios of creatinine were calculated by dividing the concentrations obtained at the start and after 60 and 240 min in dialysate by the concentrations in plasma at the same time points (D/P0, D/P60 and D/P240). A PET with an ultrafiltered volume of 400 mL or less at 4 h was considered to be significant for UFF. The sieving of Na, free water transport (FWT) and UF obtained through small pores (SPUF) was calculated as described by Bernardo AP et al. [18].

### 2.4. Antibodies

Rabbit anti-AQP1 (cat.# SC-20810), mouse anti-AQP1 (cat.# SC-25287), mouse anti-mesothelin (cat.# SC-365324) and rabbit anti-CD31 antibodies (cat.# SC-1506-R) were obtained from Santa Cruz Biotechnology (www.scbt.com). Rabbit anti-mesothelin (cat. # 14199) was obtained from Cell Signaling technology (Cellsignal.com). Mouse anti-Alix monoclonal antibodies (cat.# MA1-83977) and AlexaFluor™-conjugated secondary antibodies for immunofluorescence detection were obtained from Thermo Fisher Scientific (www.thermofisher.com). Anti-rabbit IgG whole molecules (cat.# A0545) and anti-mouse IgG H+L (cat.# 170-6516) peroxidase antibodies were purchased from Sigma-Aldrich (www.sigmaaldrich.com) and Bio-Rad (www.bio-rad.com), respectively. 

### 2.5. Immunofluorescence

Human omental tissue and parietal peritoneal samples were fixed in 4% paraformaldehyde in PBS at 4 °C; cryopreserved in 30% sucrose for 24 h; embedded in an OCT compound; and cut into 5 μm sections using a cryomicrotome. Antigen retrieval was performed by boiling sections in the citrate buffer (10 mM sodium citrate, 0.05% Tween 20, pH of 6) for 30 min. Nonspecific binding sites were blocked with 1% bovine serum albumin in phosphate-buffered saline (PBS) for 30 min at room temperature. After this, the sections were incubated overnight at 4 °C with the following primary antibodies: rabbit anti-AQP1 (1:200) and mouse anti-mesothelin (1:100). After washing in PBS, the sections were incubated with the appropriate AlexaFluor-conjugated secondary antibody (www.lifetechnologies.com) for 1 h at room temperature. After washing in PBS, the sections were mounted in PBS/glycerol (1:1) containing 1% n-propylgallate, pH 8.0. Confocal images were obtained with a confocal laser-scanning fluorescence microscope (Leica TSC-SP2, Mannheim, Germany).

### 2.6. Purification of Exosomes from Peritoneal Dialysate

The isolation of exosomes from the nocturnal peritoneal dialysate was performed using a two-step differential centrifugation method. Briefly, equal volumes of peritoneal dialysate samples were centrifuged at 12,000× *g* for 45 min at 4 °C to remove apoptotic bodies and cell debris. After this, the 12,000× *g* supernatants were ultracentrifuged at 100,000× *g* for 2 h at 4 °C. The resulting exosome-enriched pellets were resuspended in PBS and the protein concentration was determined by the Bradford assay (www.bio-rad.com).

### 2.7. Immunoblotting

A total of 15 μg of each exosome sample, which was diluted in Laemmli’s buffer with 50 mM dithiothreitol and heated for 10 min at 60 °C, was resolved on 10% SDS-PAGE before being electroblotted onto Immobilon-P PVDF membranes (www.merckmillipore.com). After blocking with 3% bovine serum albumin in TRIS buffer saline-tween 20, the blots were incubated overnight at 4 °C with the following primary antibodies: rabbit anti-AQP1 (1:500), mouse anti-Alix (1:200), mouse anti-mesothelin (1:500) and rabbit anti-CD31 (1:500). Membranes were washed and incubated with horseradish peroxidase-conjugated secondary antibodies. Reactive proteins were revealed with an enhanced chemiluminescent detection system (SuperSignal West Pico Chemiluminescent Substrate, Pierce, Rockford, IL, USA) and chemiluminescence was detected with Chemidoc XRS detection system equipped with Image Lab Software for image acquisition.

### 2.8. Immunoelectron Microscopy

For electron microscopy, the resuspended vesicle fractions were fixed in a mixture of 3% paraformaldehyde and 1% glutaraldehyde in 0.1 M PBS at a pH of 7.4 for 2 h at 4 °C. A drop of 5–10 μL of vesicle fractions were placed on clean parafilm and the grids (Formvar-carbon coated, 200 mesh Ni) were floated on the drop, with their coated side facing the suspension for 20 min. Three grids were made for each pellet preparation. The grids were transferred first to the drops of washing buffer PBS for 3 min and subsequently, to the drops of blocking buffer (BSA 1%) for 10 min. Vesicles were permeabilized with 0.1% saponin for 15 min. For immunogold labeling, the vesicle fractions were added to drops of the primary antibodies, which were mouse anti-AQP1 (1:50) or rabbit anti-AQP1 (1:100), with either rabbit anti-mesothelin (1:50) or mouse anti-Alix (1:100) antibodies overnight at 4 °C. They were washed five times using drops of washing buffer for 2 min before being incubated for 1 h at room temperature with secondary Abs, which were namely anti-mouse IgG conjugated with 5 nm gold particles or anti-rabbit IgG conjugated with 10 nm colloidal gold particles. The grids were washed using drops of PBS for 2 min before 50 μL drops of 1% glutaraldehyde were added to these grids for 5 min. Fresh drops of distillated water were used to wash these grids for 4 min. For contrast enhancement, a 50 μL drop of 2% uranyl acetate was added to these grids before the excess fluid was blotted from the grids using filter paper. The grids were observed under an electron microscope Tecnai 10 (100 Kv). For control experiments, the samples were directly incubated with the secondary Ab without pretreatment using the primary antibodies.

### 2.9. ELISA Test for AQP1 Quantitation in Peritoneal Dialysate Samples

The quantitation of AQP1 in peritoneal dialysate samples was performed using a commercial ELISA KIT (AQP1–human ELISA KIT; www.creative-diagnostics.com) with some modifications. Briefly, equal volumes of peritoneal dialysate samples (D240) from 30 patients undergoing PET (two-in-one protocol) were centrifuged at 4000× *g* for 10 min at 4 °C to remove apoptotic bodies and cell debris. After this, the supernatants were resuspended in PBS containing 1% SDS to a final concentration of 0.01% SDS. The standards and samples were added to the appropriate microtiter plate wells that were pre-coated with an antibody specific to human AQP1 and incubated for 16 h at 4 °C. On the following day, a biotin-conjugated polyclonal antibody preparation that was specific for AQP1 and Avidin conjugated to Horseradish Peroxidase (HRP) were added to each well for 1 h at 37 °C. After being washed with a washing buffer, 90 µL of the substrate solution was added to each well and incubated for 30 min in the dark at 37 °C. The enzyme substrate reaction is terminated by the addition of a sulphuric acid solution and the color change was measured spectrophotometrically at a wavelength of 450 nm. After this, the concentration of AQP1 in the samples was determined by comparing the O.D. of the samples to the standard curve and was expressed as ng/mL of the effluent.

## 3. Results

### 3.1. AQP1 Is Expressed in Human Mesothelial Cells of both Parietal and Visceral Mesothelium In Vivo

**Figure 1:**
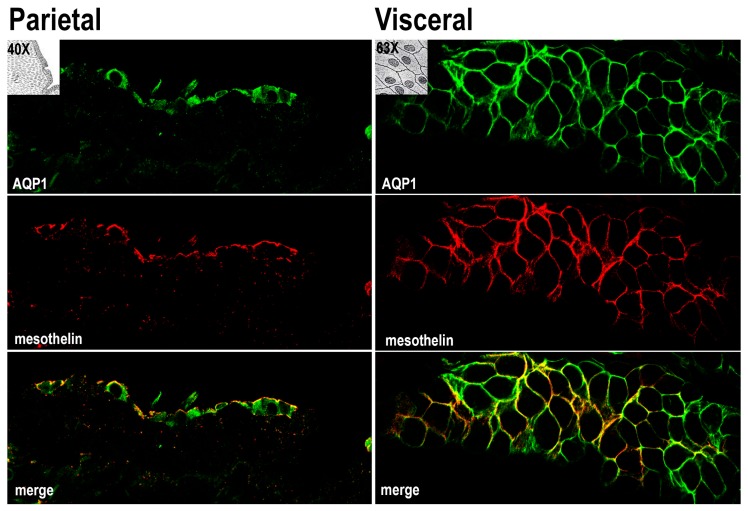
Samples of parietal and visceral (omentum) human peritoneum were analyzed by immunofluorescence with antibodies against AQP1 (green) and the surface marker of mesothelial (mesothelin) (red). Micrographs depict a longitudinal section of parietal mesothelium and a transversal section of visceral mesothelium. Colocalization (yellow) is highlighted in the merge panel. Comparable results were obtained in samples from three patients.

The samples of parietal and visceral (omentum) human peritoneum were analyzed by immunofluorescence with antibodies against AQP1 and the surface marker of mesothelial cells (mesothelin) [19]. Figure 1 depicts a longitudinal section of parietal mesothelium and a transversal section of visceral mesothelium. AQP1 (green) was localized at the plasma membrane of mesothelial cells and largely colocalized with mesothelin (merge). We also analyzed the distribution of AQP1 and the mesothelial marker CD31 in the sub-mesothelium where both antibodies are colocalized in the capillaries wall (Supplemental Figure S1). 

### 3.2. AQP1 Is Released by Mesothelial Cells in the PD Effluent through Exosomes (Qualitative Analysis) 

**Figure 2:**
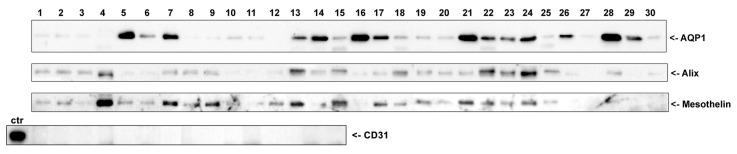
Nocturnal PD effluents from thirty PD patients were subjected to differential centrifugation to isolate an exosome-enriched fraction. Fifteen µg of total proteins from each sample were separated by SDS-PAGE and subjected to immunoblotting with antibodies against AQP1, ALIX and mesothelin. Of note, in 15 randomly chosen samples, there was no band representing the antibodies against the endothelial marked CD31 according to immunodetection. The positive control for CD31 immunoreactivity was a lysate of human omentum.

AQP1 is a membrane-spanning water channel that is constitutively localized at the cell plasma membrane in all the tissues where it is expressed [20]. Considering that in many epithelial cells, apical plasma membrane proteins can be released in the extracellular space in the form of exosomes [21], we wanted to determine if AQP1 could be released at measurable levels in the peritoneal cavity. To achieve this aim, nocturnal PD effluents from thirty PD patients were subjected to differential centrifugation to isolate an exosome-enriched fraction. As shown in Figure 2, a band at 28 kDa corresponding to AQP1 was revealed in almost all samples although this band had variable intensity. In the same samples, immunodetection showed a clear band for the exosome marker protein ALIX (apoptosis-linked gene2-interacting protein X), which is a component of the endosomal sorting complex required for transport (ESCRT) [22]. In addition, we found a variable but consistent expression of the mesothelial marker mesothelin, likely suggesting the mesothelial origin of these exosomes. Of note, there was no band depicting the antibodies against the endothelial marked CD31 [23] in the same samples according to immunodetection. 

Four randomly chosen samples of vesicles were also fixed in paraformaldehyde–glutaraldehyde, adsorbed on nickel grids and analyzed by immunogold transmission electron microscopy (TEM).

**Figure 3:**
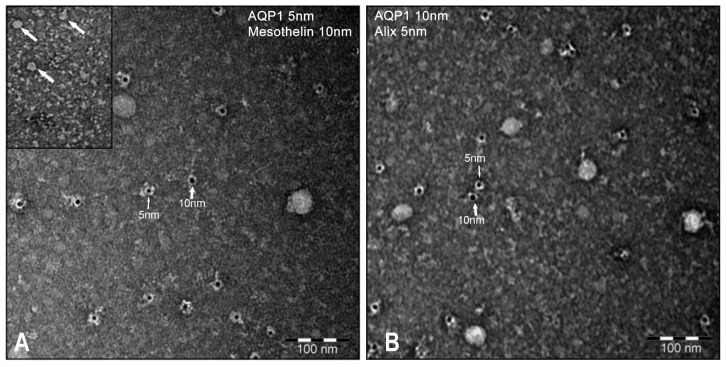
PD effluent-isolated vesicles were fixed, adsorbed on nickel grids and analyzed by immunogold transmission electron microscopy (TEM). The average size of the vesicles, which were counterstained with uranyl–acetate, was 15–20 nm. (**A**) Vesicles were decorated with 5 nm gold beads that were conjugated with monoclonal anti-AQP1 antibodies and with 10 nm gold beads that were conjugated with polyclonal anti-mesothelin antibodies (left panel). (**B**) Vesicles were also decorated with 5 nm gold beads that were conjugated with monoclonal anti-Alix antibodies and with 10 nm gold beads that were conjugated with polyclonal anti-AQP1 antibodies (right panel). Comparable results were obtained in all vesicle samples.

As shown in Figure 3, the average size of the vesicles, which were counterstained with uranyl–acetate, was 15–20 nm. Vesicles were decorated with 5 nm gold beads that were conjugated with monoclonal anti-AQP1 or Alix antibodies and with 10 nm gold beads that were conjugated with polyclonal anti-mesothelin or AQP1 antibodies. Unfortunately, due to the very small size of the vesicles, we could not detect vesicles that were colabelled by AQP1 and one of the other markers. This may be likely due to the steric hindrance between gold particles. Comparable results were obtained in all vesicle samples. The same results were obtained when the exosomes were isolated with the exosome isolation reagent ExoQuick-TC (System Biosciences, www.systembio.com; not shown). 

### 3.3. AQP1 Abundance in the PD Effluent Positively Correlates with the Efficiency of PD (Quantitative Analysis)

**Figure 4:**
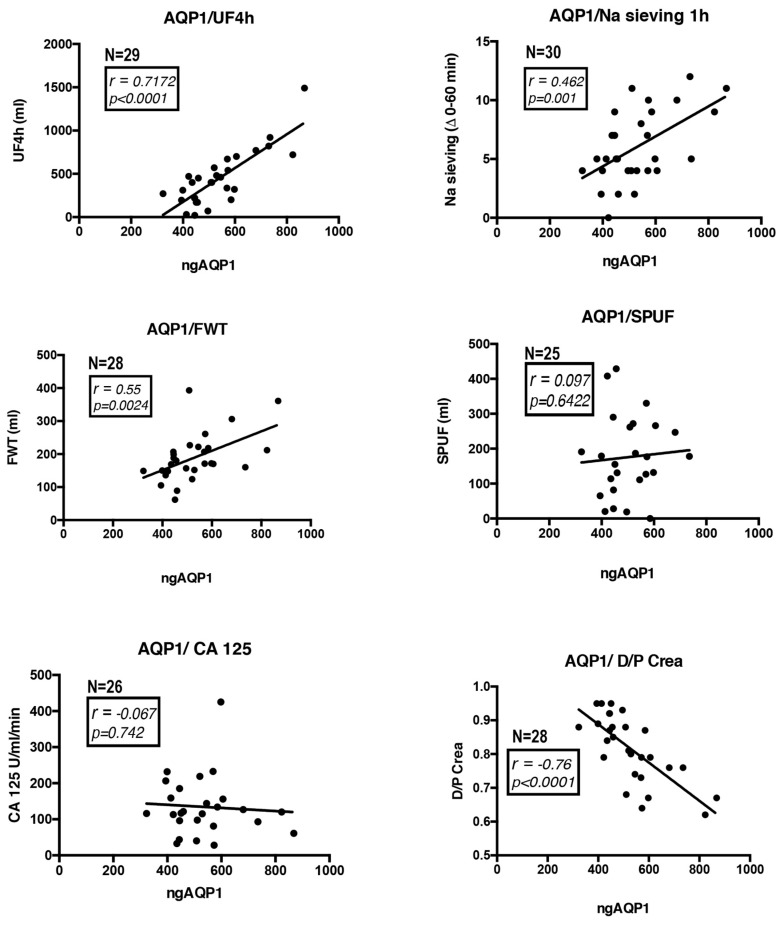
AQP1 was quantified in PD effluent samples from patients undergoing a 4-h, 3.86% glucose PET. AQP1 abundance, which was expressed as the amount of AQP1 in nanograms in the total volume (ngAQP1) of PD effluent drained at the end of the PET test, was correlated with ultrafiltration (UF), sodium (Na) sieving, free water transport (FWT), small-pore ultrafiltration (SPUF), Cancer Antigen 125 (CA125) and the dialysate-to-plasma ratios of creatinine (D/P Crea). Correlation analysis was performed assuming non-Gaussian distribution of the data and calculating nonparametric Spearman correlations. The number of patients analyzed (N) is indicated in each panel.

We analyzed thirty PD effluent samples from patients undergoing a 4-h, 3.86% glucose PET with additional measurement of ultrafiltration (UF), sodium (Na) sieving, free water transport (FWT), small-pore ultrafiltration (SPUF), Cancer Antigen 125 (CA125) and the dialysate-to-plasma ratios of creatinine (D/P Crea). AQP1 was quantified in all samples using a commercial AQP1 ELISA kit specific for the high sensitivity detection of human AQP1 in biological fluids. AQP1 abundance, which was expressed as the amount of AQP1 in nanograms in the total volume of PD effluent drained at the end of the PET test, was correlated with all the above parameters describing the efficiency of the PD. Correlation analysis was performed assuming a non-Gaussian distribution of the data and calculating nonparametric Spearman correlations. The analysis results are shown in Figure 4. AQP1 abundance in the PD effluent was positively and significantly correlated with both UF (*r* = 0.7172, *p* < 0.0001), Na sieving (*r* = 0.462, *p* < 0.01) and FWT (*r* = 0.55, *p* < 0.01). AQP1 abundance in the PD effluent are correlated neither with the SPUF nor with the CA125. A negative, highly significant correlation was found between AQP1 abundance and the D/P Crea (*r* = −0.76, *p* < 0.0001). 

## 4. Discussion

In this present work, we provide the first experimental evidence that the water channel AQP1 that is expressed by mesothelial cells lining the peritoneal membrane is released in the PD effluent using exosomes and is positively correlated with the efficiency of PD.

During PD, the pathways that are available for solute and water exchange between the peritoneal capillaries and the hyperosmolar solution in the peritoneal cavity is characterized by the presence of the sequence of (a) a continuous capillary endothelium, (b) the peritoneal interstitium and (c) the mesothelium. 

Using a computational simulation, Rippe et al. [7,24] first suggested that the peritoneal membrane permeability is best described by a three-pore model. They considered the capillaries endothelium as the rate-limiting barrier to the solute exchange between the capillaries and the peritoneal cavity. In the capillary wall, rare transendothelial pores (referred to as large pores, up to 250 Å) [25] allow the transport of macromolecules and when combined together with the interendothelial cleft (small pores, up to 40 Å), this allows the transport of low-molecular-weight solutes, such as glucose, urea and creatinine. An “ultra-small, water-only” pore (3 to 5 Å) has been postulated to be selective for water and responsible for the majority of osmotically induced water transport [26]. 

The discovery of the aquaporin (AQP) family of water-channel proteins (see [27,28] for review) provided new insights into the molecular mechanisms of the transcellular water movement across the peritoneum. An abundant expression of the AQP1 isoform was reported to be found in the endothelial cells of peritoneal capillaries and venules but not in arterioles or mesothelial cells by immunocytochemistry [13]. In a subsequent study, AQP1 was also found in human mesothelial cells and upregulated by exposure to the osmotic agents, namely glucose and mannitol [14]. The analysis of AQP1 knockout mice demonstrated that AQP1 provides a major route for osmotically driven water transport across the peritoneal barrier in peritoneal dialysis [29]. In contrast, AQP1 deletion had little effect on slow isosmolar fluid absorption from the peritoneal cavity. More recently, novel endothelial cell-specific AQP1 knockout mice confirm the crucial functional role of endothelial AQP1 in ultrafiltration during PD [30].

Our observation that AQP1 is abundantly expressed at the plasma membrane of mesothelial cells in both parietal and visceral peritoneum biopsies (Figure 1) prompted us to investigate whether AQP1 could be released in the peritoneal cavity through exosomes and are subsequently expelled in the PD effluent. Exosomes are vesicles composed of membranes that are released into the extracellular environment upon the exocytic fusion of multivesicular endosomes with the cell surface [31]. In this work, we provide compelling evidence that an exosome-enriched fraction obtained from PD dialysates is enriched in both the water channel AQP1, the exosome marker Alix [32] and the mesothelial marker mesothelin [33] but devoid of the endothelial marker CD31 [23] (Figure 2). This result would support the mesothelial origin of the AQP1 released into the peritoneal cavity. In addition, even if AQP1 was excreted in exosomes by the endothelial cells of the peritoneal capillaries, these vesicles could not make it to the peritoneal cavity since they would be stuck by the interstitial collagen bundles and could not pass the barrier formed by mesothelial cells. Transmission electron microscopy showed that the high-speed pellet isolated from PD dialysates is mainly composed by small, round shaped, membrane delimited structures with a homogeneous size compatible with that of exosomes (Figure 3) [34]. Immunogold staining of these exosomes showed that they were positive for AQP1. In fact, two different anti-AQP1 antibodies, one of which was monoclonal (Figure 3A, 5 nm gold particles) and the other polyclonal (Figure 3B, 10 nm gold particles), were able to stain many of these vesicles. Exosomes were also stained by anti-mesothelin antibodies (Figure 3A, 10 nm gold particles) and Alix (Figure 3B, 5 nm gold particles). Unfortunately, the small size of the exosomes (~20 nm on average) did not allow the colocalization of AQP1 neither with Alix nor with mesothelin. This is likely because the 10 and 5 nm immunogold particles could not fit on the same vesicle. The observation that the size of the immunostained vesicles was homogeneous and that they were stained by all the three antibodies suggests that they are exosomes with an mesothelial origin that expresses AQP1. 

It has been previously demonstrated that in the kidney tubule, the water channel AQP2 is released by epithelial cells in the tubular space through exosomes that can be isolated from the urine [21]. Interestingly, the amount of urinary AQP2 is proportional to the amount of AQP2 expressed at the plasma membrane of renal cells. Therefore, urinary AQP2 is considered to be a reliable noninvasive biomarker for evaluating a number of physiopathological conditions affecting the urine concentrating ability of the kidney [35,36,37,38,39]. 

Therefore, we hypothesized that the amount of excreted AQP1 in the PD effluent could reflect the amount of AQP1 expressed by mesothelial cells. Thus, this could act as a biomarker of the health status of the peritoneal membrane in PD patients. Strikingly, we show here that the amount of AQP1 released in the PD effluent was nicely correlated with the efficiency of the PD. In fact, AQP1 abundance in the PD effluent was positively correlated with the ultrafiltration (UF) calculated during the PET test. The pivotal role of AQP1 expressed in the peritoneal capillary endothelium in mediating UF was clarified by Ni et al. [40], which demonstrated that AQP1^-/-^ mice had a reduction of nearly 50% in UF during an adapted PET test. For the first time, we also showed that the amount of mesothelial AQP1 released into the PD effluent was positively correlated with the net UF during the first hour of a PET test in humans. Sodium sieving represents the fall in dialysate sodium concentration during a dwell with hypertonic glucose and it strictly depends on water UF. In our study, we showed that the amount of AQP1 in the PD effluent was positively and significantly correlated with the Na sieving during the first hour of the PET test. During the first hour of a modified PET, a strong osmotic gradient over AQP1 induces free water transport (FWT) from the capillaries to the dialysate, resulting in a decrease of the dialysate sodium concentration. Hence, this dip in dialysate sodium is an indirect method for estimating the magnitude of water transport through the ultra-small pores [18]. FWT, which was calculated in our study during the first hour of dwell, was also positively correlated with the amount of the ultra-small pore AQP1 in the PD effluent. Conversely, as expected, AQP1 in the effluent was not correlated with the small pore ultrafiltration (SPUF). Moreover, we showed that effluent AQP1 is not correlated with the amount of effluent CA125 which is a marker of mesothelial cell mass that is currently taken into account in clinical practice [41]. 

Taken together, these data strongly suggest that both endothelial AQP1 and mesothelial AQP1 might have a functional role in mediating solute-free water transport in PD. The predictive three-pore model does not take into account the possible contribution of the mesothelial layer as a selective barrier to the osmotic diffusion of water during PD [7,10,24,25,42]. The generation of endothelial-specific AQP1 knockout mice [30] confirmed the prediction of the computational model by Rippe & Stelin but did not add information about the functional role of mesothelial AQP1 in PD. It is often concluded that mesothelium does not act as a functional barrier during PD. However, the intact peritoneal mesothelial cells display a well-developed microvillus border (see [43] for review) and most importantly tight junctions or zonulae occludentes [44,45], which contribute to the establishment and maintenance of a continuous epithelium-like cell monolayer and reduce transcellular water permeability [46]. The abundant expression of AQP1 in mesothelial cells, along with the fact that its expression is upregulated in vitro by hypertonic glucose-based solutions [14], suggests a functional role of mesothelial AQP1 in mediating transmesothelial water movement during PD. Our findings of the amount of AQP1 excreted by the mesothelium during PD being correlated with the efficiency of PD, as assessed by PET, suggest that the continuous monitoring of AQP1 levels in the PD effluent may represent a strategy to predict a decrease in PD efficiency and UFF. The method of quantitation that is based on an ELISA test is rapid and has a low cost. Furthermore, this requires only a microvolume of PD effluent and importantly, this test is non-invasive for the patient. Therefore, we propose AQP1 as a non-invasive biomarker reflecting both the health and the function of the peritoneal barrier in PD patients. 

We are aware that further research on a larger group of patients and a better characterization of AQP1-positive vesicles would help to strengthen our conclusion.

## Supplementary Materials

The following are available online at https://www.mdpi.com/2073-4409/8/4/330/s1.
