# Supplementary material for: AQP1-Containing Exosomes in Peritoneal Dialysis Effluent As Biomarker of Dialysis Efficiency
Source: Cells. 2019 Apr 9;8(4):330. doi: 10.3390/cells8040330 (PMC6523141; doi:10.3390/cells8040330)
Supplement: Supplementary file 1 [file cells-08-00330-s001.pdf]

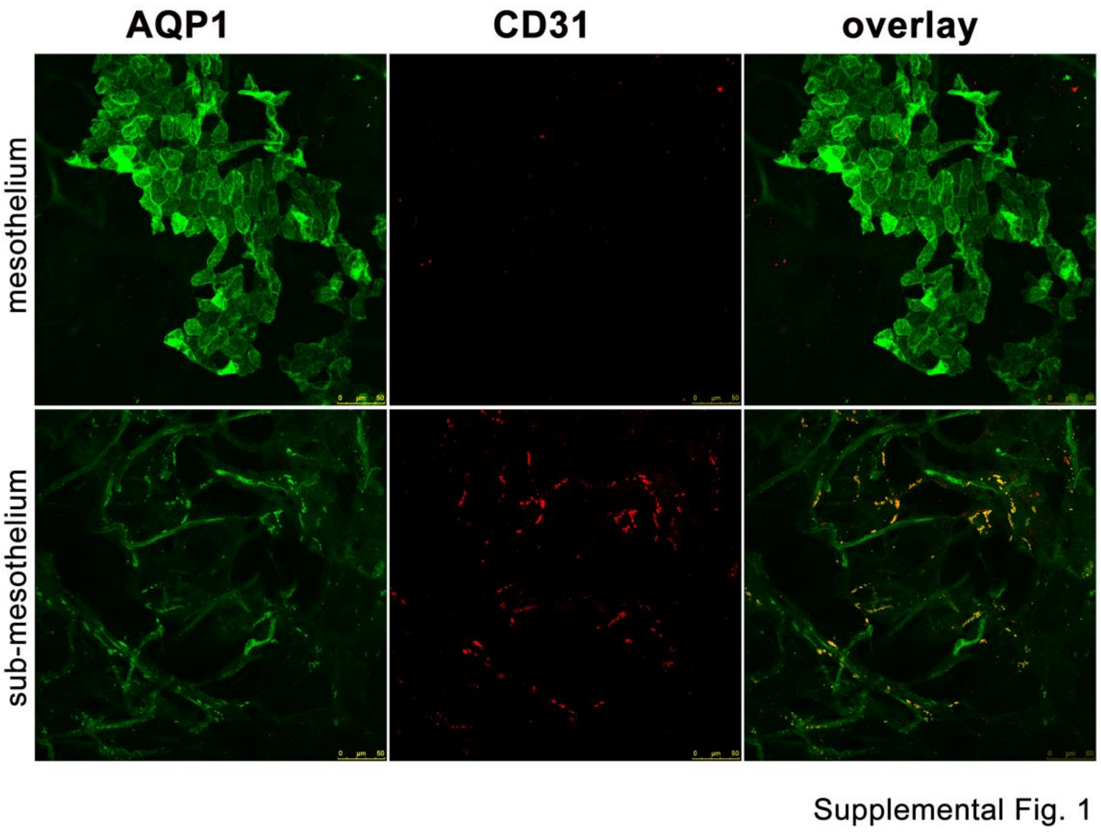

Supplemental Figure. S1 Human omental biopsies were stained with anti AQP1 antibodies (green) and anti-CD31 antibodies (red). In the mesothelial layer, AQP1 stained the plasma membrane of mesothelial cells that were negative for CD31 expression. A confocal scan taken in the submesothelial space showed co-localization of AQP1 and CD31 in the capillaries.
